# Spatial proteomics to discover aging-associated alterations in the renal tubulointerstitium

**DOI:** 10.1186/s12014-025-09550-8

**Published:** 2025-10-08

**Authors:** Dong-Gi Mun, Ganesh P. Pujari, Gunveen Sachdeva, Benjamin J. Madden, M. Cristine Charlesworth, Kenneth L. Johnson, Luisa Ricaurte Archila, Mariam P. Alexander, Aleksandar Denic, Aidan F. Mullan, Vidit Sharma, Nicholas B. Larson, Anthony C. Luehrs, Andrew D. Rule, Akhilesh Pandey

**Affiliations:** 1 Department of Laboratory Medicine and Pathology, Mayo Clinic, 200 First Street SW, Rochester, MN 55905 USA; 2 Manipal Academy of Higher Education, Manipal, 576104 Karnataka India; 3 Proteomics Core, Mayo Clinic, Rochester, MN 55905 USA; 4 Division of Nephrology and Hypertension, Mayo Clinic, 200 First Street SW, Rochester, MN 55905 USA; 5 Division of Clinical Trials and Biostatistics, Mayo Clinic, Rochester, MN 55905 USA; 6 Department of Urology, Mayo Clinic, Rochester, MN 55905 USA; 7 Division of Epidemiology, Mayo Clinic, Rochester, MN 55905 USA; 8 Center for Individualized Medicine, Mayo Clinic, Rochester, MN 55905 USA

**Keywords:** Spatial proteomics, Renal aging, Tubulointerstitium

## Abstract

The preservation of tissue architecture and morphology in formalin-fixed paraffin-embedded (FFPE) tissues enables spatial resolution at the cellular and sub-cellular levels. Laser capture microdissection (LCM) combined with liquid chromatography tandem mass spectrometry analysis permits collection of tissue areas with spatial context for proteome profiling from FFPE slides. In this study, we performed proteome profiling of non-diseased renal tubulointerstitial tissue in a cohort of young (< 40 years) and old (> 70 years) individuals with the goal of spatially correlating the histomorphology to the proteomic profile. To perform in-depth characterization of renal tubulointerstitium and to identify renal aging-associated proteins, a multiplexing strategy using tandem mass tags (TMT) was employed, resulting in the quantitation of 7,355 proteins. Our approach allowed for identification of proteins with low abundance such as fibrocystin and ninein-like protein. Notably, 162 solute carrier proteins from 47 solute carrier families were identified, which were enriched in proximal and distal tubule cells. Finally, we discovered a proteomic signature associated with renal aging, which includes metalloproteinase inhibitor 3, nicotinamide N-methyltransferase, matrix metallopeptidase 7, phenazine biosynthesis-like domain-containing protein and solute carrier family 23 member 1. Overall, our study demonstrates the power of LCM combined with proteomics to leverage archived FFPE tissue samples for investigating proteomic alterations in the renal tubulointerstitium with age at a high depth of proteome coverage.

**Supplementary Information:**

The online version contains supplementary material available at 10.1186/s12014-025-09550-8.

## Introduction

The ability to quantify the spatial distribution of different proteins across wide sections of intact tissue at subcellular precision is essential for understanding how the phenotype of individual cell links to the function of the multicellular structures they comprise. With histopathological evaluation of tissue sections being a standard of care for diagnosis and prognosis of many disorders, formalin-fixed paraffin-embedded (FFPE) tissue blocks are the most comprehensive resource available for clinical research. FFPE blocks while being an invaluable resource, have inherent challenges for proteomic analysis because of formation of crosslinks between proteins due to formalin fixation [1]. However, there has been considerable research and experimentation to maximize protein recovery by borrowing methodology such as heat induced antigen retrieval from immunohistochemical practices, the efficacy of which have been validated by comparison of recovery from FFPE sections and fresh frozen tissue [2].

LCM combined with proteomics using liquid chromatography-mass spectrometry (LC-MS/MS) has enabled deep proteome profiling in both health and disease while preserving relevant spatial biological information [3, 4]. In this study, we harnessed the power of LCM sampling to investigate the changes in the tubulointerstitial compartment with age at spatial resolution. The study of structural and functional changes in the kidneys with aging requires distinguishing changes from chronic kidney diseases from changes due to the senescence associated phenotype [5]. At the microscopic level, the primary aging-related changes in the kidney parenchyma are nephron loss from nephrosclerosis with focal interstitial fibrosis and tubular atrophy. This atrophy and eventual loss of nephrons results in loss of kidney cortical volume at the macroscopic level. Identifying proteins that are differentially expressed with aging in normal tubules may lead to better understanding of the aging process in kidneys. We deployed a labeling strategy using tandem mass tag (TMT) for deep proteome coverage of LCM samples, which resulted in quantitation of 7,355 proteins across 16 samples with identification of kidney specific proteins functioning as transporters, transmembrane receptors and ion channels. Finally, we identified a proteomics signature related to renal aging at spatial resolution in the normal tubules and interstitium excluding regions of interstitial fibrosis and atrophic tubules.

## Results and discussion

### Clinical characteristics of samples

For an in-depth characterization of the aging tubulointerstitial proteome, the relevant patient demographics and kidney characteristics for the sixteen patients who underwent a radical nephrectomy for kidney tumor (with no cancer recurrence for at least 3 months) were included in this study as summarized in Table 1 and Supplementary Table 1. As we were interested in the proteome of aging rather than chronic kidney disease and the early proteomic changes that preceded sclerotic/fibrotic changes [6, 7], we only studied patients with < 5% globally sclerotic glomeruli (GSG) and < 5% interstitial fibrosis and tubular atrophy (IFTA) on standard histology. Patients were grouped based on age, with patients under 40 years old at surgery comprising the “young” cohort (mean age 29 years, *n* = 8) and patients aged 70 or older at surgery comprising the “old” cohort (mean age 79 years, *n* = 8). Baseline clinical characteristics were obtained from medical records at the time of the nephrectomy (Table 1). Older patients differed from younger patients in having smaller kidneys with reduced cortical and medullary volume, a lower percentage of non-sclerosed glomeruli, a higher percentage of IFTA, and more luminal stenosis.

**Table 1:** Summary of young and old matched nephrectomy patients

Characteristics	Young (*N* = 8)	Old (*N* = 8)	*p*-value
Age, years	29.0 (4.9)	79.3 (5.3)	---
Sex			> 0.99
Female	3 (38%)	4 (50%)	
Male	5 (63%)	4 (50%)	
Race			> 0.99
White	7 (88%)	8 (100%)	
Non-White	1 (13%)	0 (0%)	
BMI	25.2 (3.4)	24.4 (3.2)	0.40
Hypertension	0 (0%)	4 (50%)	0.077
Diabetes Mellitus	0 (0%)	0 (0%)	> 0.99
Smoking History	3 (38%)	4 (50%)	> 0.99
Kidney Volume, cm^3^			
Total Volume	172.5 (52.9)	146.9 (34.6)	0.36
Cortex	124.5 (39.1)	105.6 (21.7)	0.30
Medulla	48.0 (14.1)	41.3 (14.2)	0.30
Microstructural Characteristics			
NSG Density, per mm^3^	25.1 (7.8)	18.1 (5.8)	0.036
Cortex volume per NSG, mm^3^	0.043 (0.014)	0.058 (0.019)	0.035
Non-Fibrotic cortex volume per NSG, mm^3^	0.043 (0.014)	0.059 (0.019)	0.040
Mean tubular profile area, µm^2^	4,379 (1,285)	5,287 (1,047)	0.093
Percent GSG, %	1.0 (1.1)	3.6 (0.9)	0.003
Percent luminal stenosis, %	36.8 (8.0)	61.5 (11.4)	0.001
Percent IFTA, %	0.1 (0.1)	1.3 (0.8)	< 0.001
Mean IFTA focus area, µm^2^	25,320 (18,867)	66,915 (32,781)	0.005

BMI = body mass index, NSG = non sclerotic glomerulus, GSG = globally sclerotic glomerulus, IFTA = interstitial fibrosis and tubular atrophy. P-value was calculated using Fisher’s exact test

### In-depth proteome coverage of LCM FFPE samples using multiplexed TMT-approach

**Fig. 1:**
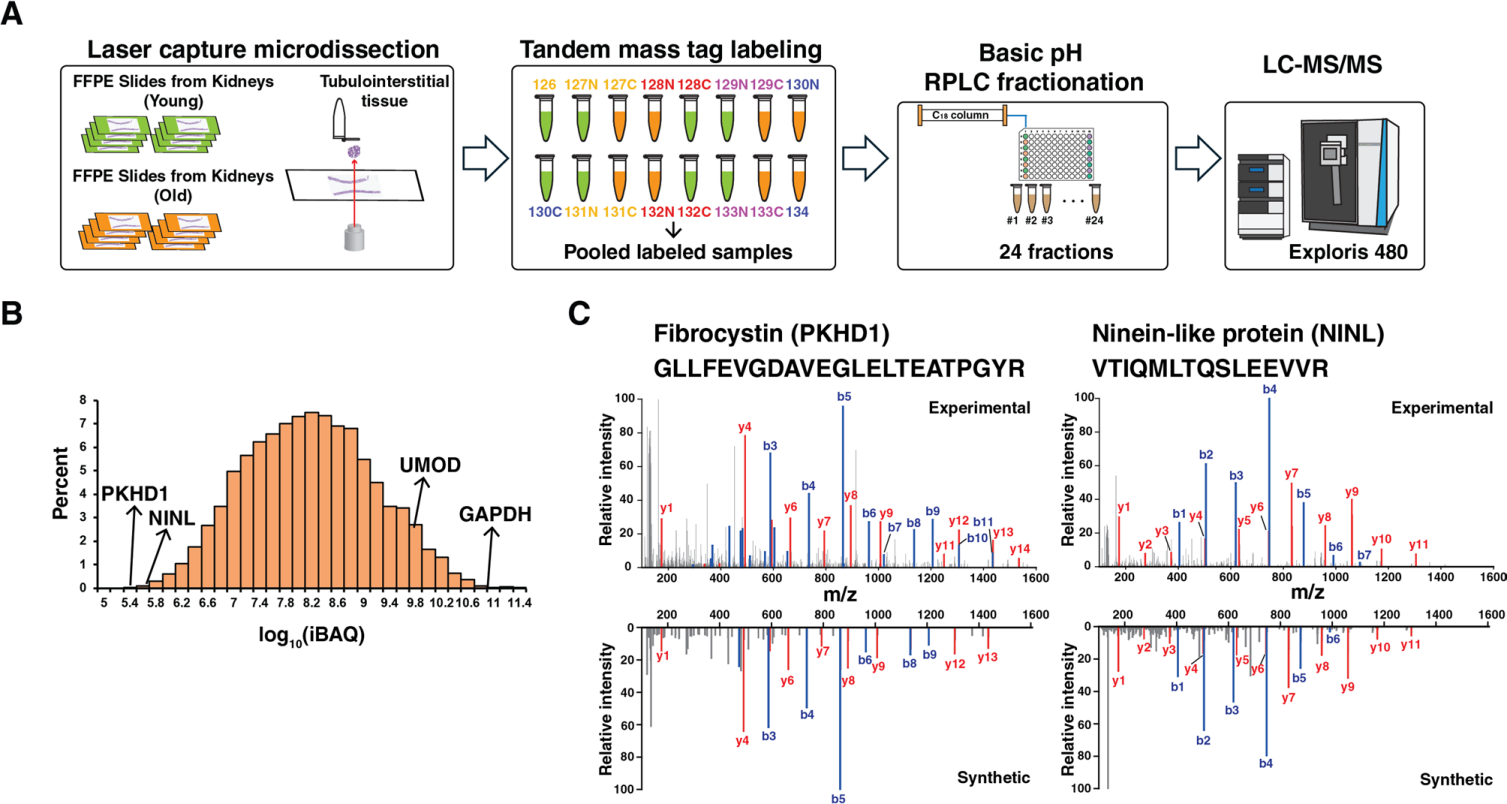
Overview of proteome analysis of renal formalin-fixed paraffin-embedded (FFPE) tissues using laser capture microdissection. (**A**) Sample preparation workflow for proteomics analysis of FFPE tissues. FFPE slides from kidney of young (*n* = 8) and old (*n* = 8) group were used. Two 10 μm serial sections from FFPE tissue on PEN-membrane were used for the study. After protein extraction and digestion, equal amount of peptides (40 µg per each) were labeled using 16-plex TMT reagents and fractionated into 24 fractions. Samples were analyzed using Orbitrap Exploris 480 mass spectrometer. (**B**) Protein intensity distribution estimated by intensity-based absolute quantification (iBAQ) value plotted in log_10_ scale. Selected proteins with high and low iBAQ values. (**C**) Annotated MS/MS spectra of peptide GLLFEVGDAVEGLELTEATPGYR from fibrocystin and VTIQMLTQSLEEVVR from ninein-like protein. MS/MS spectra of corresponding synthetic peptides are shown as mirror plots

We employed laser capture microdissection of FFPE kidney parenchymal sections (away from the tumor), tandem mass tagging with offline high pH HPLC fractionation and LC-MS/MS to compare the proteomic profiles of the deep tubulointerstitium from 8 young (< 40 yrs) and 8 old (> 70 yrs) matched nephrectomy patients (Fig. 1A). Two serial sections of 10 μm thickness from the FFPE renal tissue for each individual were used for the study. The sections were deparaffinized and an expert pathologist annotated the tubulointerstitial regions of interest from the deeper compartments of the cortex excluding glomeruli. Areas of IFTA were excluded from this annotation based on histological evaluation prior to LCM. Such areas were excluded in order to identify proteins associated with aging in the normal-appearing tubulointerstitium. LCM was performed for renal tubulointerstitium tissues followed by protein lysis and digestion, which resulted in peptide amounts ranging from 45 µg to 133 µg.

To perform TMT labeling, 40 µg of peptide material from each sample was reacted with tandem mass tag regents. To reduce the sample complexity and increase protein identification coverage, we used offline basic pH reversed-phase high pressure liquid chromatography and separated the peptides into 96 fractions which were subsequently concatenated into 24 samples for analysis with nanoLC high resolution mass spectrometry. This approach resulted in identification of 89,607 peptides from 7,355 proteins. We first evaluated the abundance of identified proteins in the samples by calculating intensity-based absolute quantification (iBAQ), which was determined by dividing the sum of the quantified peptide intensities by the number of theoretical tryptic peptides [8]. This measure of protein abundance revealed that the abundance of identified proteins spanned ~ 6 orders of magnitude with the median iBAQ of 8.1 on a log_10_ scale (Fig. 1B). Proteins such as glyceraldehyde-3-phosphate dehydrogenase (GAPDH) and uromodulin (UMOD), which are known to be expressed in the kidney, were abundant. Glyceraldehyde-3-phosphate dehydrogenase is a housekeeping protein and has been predominantly used as an endogenous control for kidney study [9]. Uromodulin, produced by the kidneys, is among the most abundant proteins in urine with its levels indicating renal tubular function [10]. In addition, we confidently identified low abundance proteins as shown in the annotated MS/MS spectra for peptides derived from two representative low abundance proteins, fibrocystin (PKHD1) (iBAQ value 5.3) and ninein-like protein (NINL) (iBAQ value 5.5) (Fig. 1C and Supplementary Fig. 1). As these proteins were identified with one peptide, we further confirmed their identification using synthetic peptides (Fig. 1C). Interestingly, fibrocystin is known to be expressed in the epithelial cells of renal tubulointerstitial tissue [11]. While proteomic analysis of kidney FFPE tissues using LCM has been investigated previously, the majority of studies have focused on glomeruli in the context of glomerular disease [12–14]. A recent study using laser captured renal tubular tissue from individuals with autoimmune related kidney diseases identified an average of 2,200 proteins from 5 to 8 tubules per individual [15]. Another study of IgA nephropathy focusing on microdissected tubulointerstitial tissue of ~ 3 million µm^2^ resulted in 2,562 proteins [16]. To the best of our knowledge, this is the first study to obtain an in-depth proteome coverage from renal tubulointerstitial tissues obtained by LCM. In addition, while most studies were label-free based quantitation, we employed TMT-based multiplexing on LCM samples for improved quantitation.

### Exploring the renal tubulointerstitium tissue proteome from microdissected samples

**Fig. 2:**
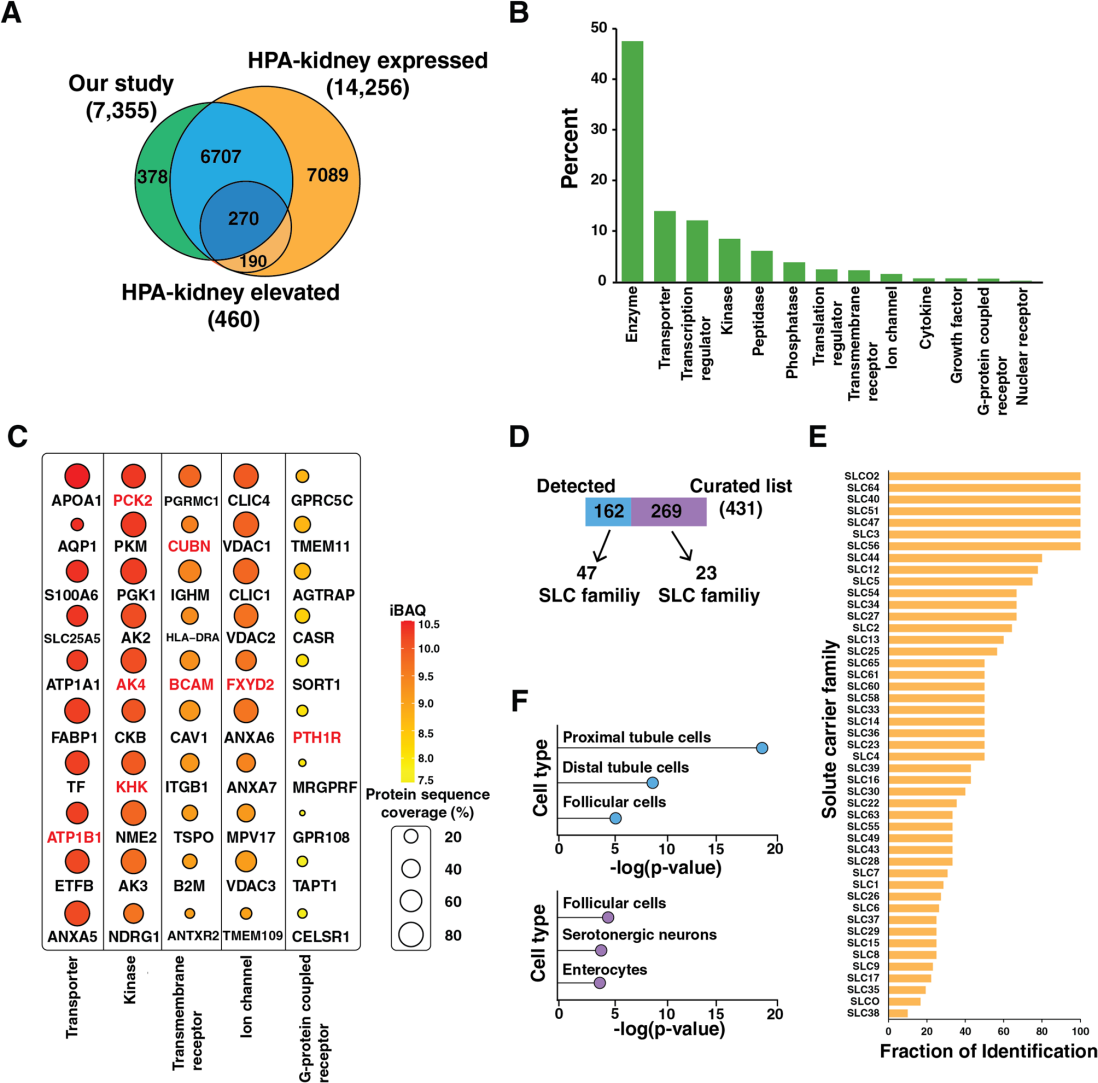
Proteome coverage of renal tubulointerstitium after LCM. (**A**) Comparison to the proteins in human protein atlas. Proteins expressed in the kidneys and those showing elevated expression were obtained from the Human Protein Atlas (https://www.proteinatlas.org/humanproteome/tissue/kidney). (**B**) Distribution of molecular types of identified proteins. **(C)** Top 10 proteins of several types of molecules (transporter, kinase, transmembrane receptor, ion channel and G-protein coupled receptor) are shown with their intensity-based absolute quantification (iBAQ) value and protein sequence coverage. Proteins with kidney-elevated expression are highlighted in red. (**D**) Comparison with a curated list of solute carrier family proteins (available at https://esbl.nhlbi.nih.gov/Databases/SLC-list). (**E**) Fraction of identification of 47 SLC family. (**F**) Cell type enrichment analysis of 162 and 269 SLC proteins against Panglao database using Enrichr (https://maayanlab.cloud/Enrichr)

The proteome profiles led us to evaluate the characteristics of the identified proteins from LCM samples of renal tubulointerstitial tissues. As expected, most of identified proteins (95%) were reported in the human protein atlas (HPA). Of these, we identified 270 proteins with kidney specific expression classified according to the HPA [17] (Fig. 2A). Functional annotation of the identified proteins revealed that we identified various classes of molecules including 552 transporters and 59 ion channel proteins (Fig. 2B). Given that we analyzed microdissected sample from FFPE slides, we further explored the types of proteins within molecular categories associated with renal function including transporter, kinase, transmembrane receptor, ion channel and G-protein coupled receptor (Fig. 2C). Several proteins with elevated expression in kidney such as sodium/potassium-transporting ATPase subunit beta-1 (ATP1B1), phosphoenolpyruvate carboxykinase 2 (PCK2), cubilin (CUBN) and parathyroid hormone 1 receptor (PTH1R) are already shown among top 10 proteins for each molecular type. The entire list of proteins is summarized in Supplementary Table 2 with their iBAQ values and molecular types, which we believe will provide valuable information of tubulointerstitium tissue proteome at spatial resolution.

One of the major functions of renal tubules is transport at both the apical and basolateral tubular cell membrane via transporters, which include ATPases, ion channels and solute carriers. We further explored whether we detected solute carrier (SLC) transporters in histologically normal renal tubulointerstitial tissue of the cortex. The 7,355 identified proteins were compared to publicly available curated list of 431 SLC proteins of the kidney from 70 SLC families [18], revealing an overlap 162 proteins (Fig. 2D). We covered an average of 50% of each SLC family proteins (Fig. 2E). The majority were proteins from the SLC25 family, in which 30 proteins were identified out of 53 proteins in the curated list. Cell type enrichment analysis of 162 proteins using Panglao database [19] showed that proximal and distal tubule cells were highly ranked, which supports that our approach identified SLC proteins specific to the renal tubulointerstitium tissue. In addition, 269 proteins not identified in our study were not enriched in tubule cells (Fig. 2F).

Mass spectrometry-based proteomics has already been widely deployed to study FFPE tissue [20, 21]. Through technologies such as LCM, samples with spatial resolution for specific microscopic structures are now being increasingly investigated with development of highly sensitive mass spectrometry [22–25]. Here, we were able to exploit the power of TMT-based multiplexed quantitation in the context of LCM-based spatial proteomics to specifically obtain greater depth of renal tubulointerstitial tissue proteome.

### Identification of proteins potentially related to renal aging

**Fig. 3:**
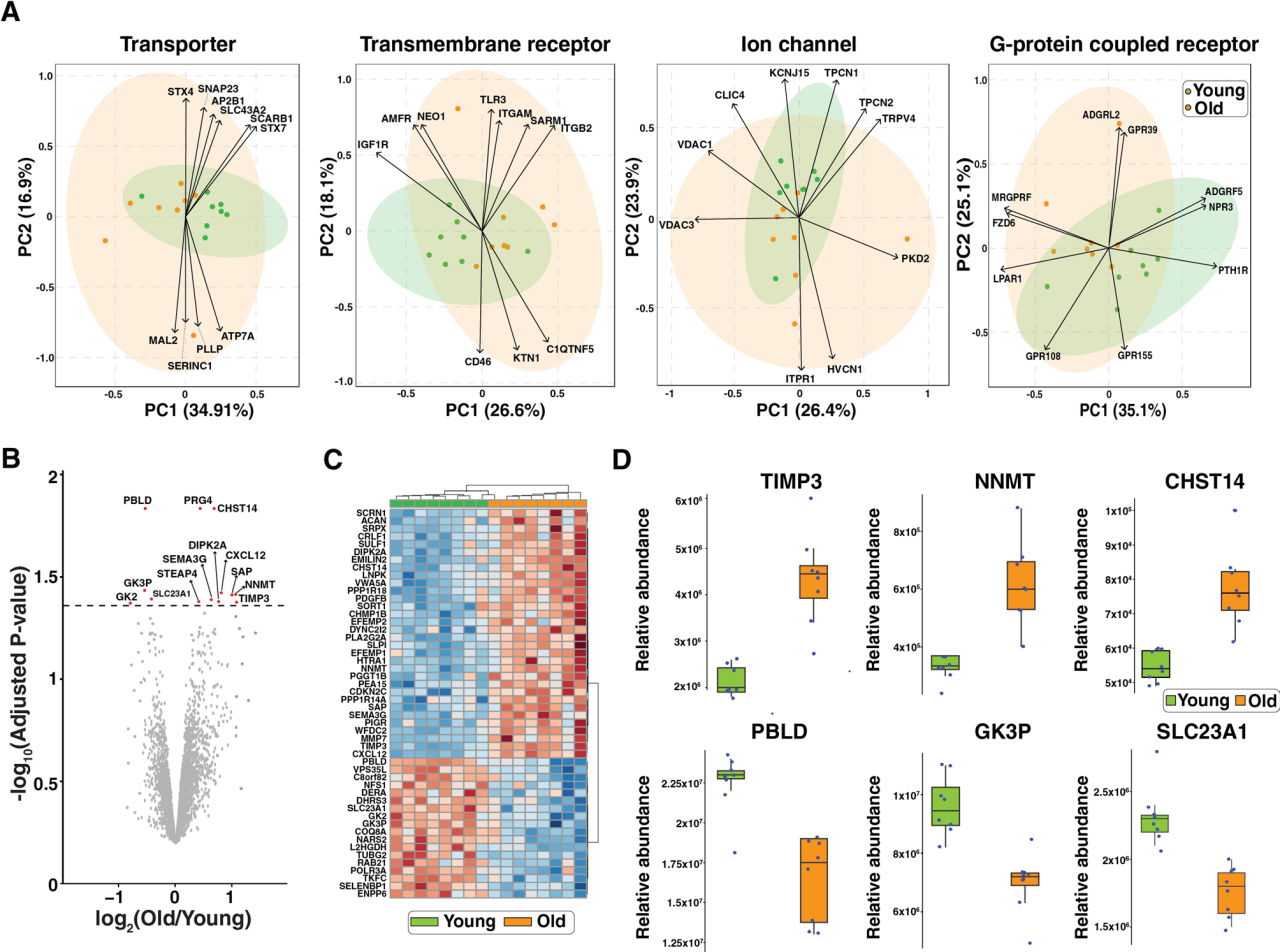
Proteomic changes in the renal tubulointerstitium with aging. (**A**) Biplot of principal component analysis overlaying score plot and loading plot (top 10 proteins). PCA was performed for groups of proteins of transporter, transmembrane receptor, ion channel and G-protein coupled receptor. (**B**) A volcano plot showing the differentially expressed proteins detected comparing renal tubulointerstitium from the FFPE tissue of old patient cohort (*n* = 8) with the young (*n* = 8). Significantly differentially expressed proteins are highlighted in red (adjusted p-value < 0.05). (**C**) A heat map displaying the Z-score of top 50 differentially expressed proteins. (**D**) Box plots showing distribution of representative differentially expressed proteins with their relative abundance in the young (green) and old (orange) cohorts

To explore the differences between old and young cohort, we compared the protein abundance of 7,355 proteins, which were identified and quantified from all samples (Supplementary Table 2). First, principal component analysis was performed for proteins grouped by their functions of transporter, transmembrane receptor, ion channel and G-protein coupled receptor (Fig. 3A). In general, we observed a good separation between young and old groups. In addition, we investigated the top ten proteins that contribute to such separation. Notably, several proteins were reported to play a role in aging and kidney disease. For example, copper-transporting ATPase 1 (ATP7A) is reported as expressed in proximal and distal tubules with age-dependent localization from mice study [26]. Decreased parathyroid hormone 1 receptor (PTH1R) expression is known to be related to renal failure [27, 28]. In addition, the overexpression of lysophosphatidic acid receptor 1 (LPAR1) expressed in tubular epithelial cells has been reported to be associated with chronic inflammation and renal fibrosis with increased expression with age from mice study [29].

Next, we performed differential protein expression analysis using multivariate regression analysis, which revealed that 9 proteins were significantly increased, and 4 proteins were decreased in the old group (adjusted p-value < 0.05) (Fig. 3B). The hierarchical clustering of top 50 proteins differentiating two groups of young and old revealed proteins with clear differences in abundance between the two groups (Fig. 3C). In addition, box plots of representative differentially expressed proteins clearly highlighted significant changes abundance with aging. Notably, some of the differentially expressed proteins have previously been studied for their roles in aging although not necessarily in the context of human renal tubulointerstitium. For example, an age-dependent increase in the abundance of tissue metalloproteinase inhibitor 3 (TIMP3) was evidenced by development of tubulointerstitium fibrosis in TIMP3 mutated aging mice [30, 31]. TIMP3 deletion in mice results in an overexpression of type I, type III collagen and TGF-b, suggesting that increase of TIMP3 protein in tubulointerstitium may be an additional defense against TGF-b-induced fibrosis. Nicotinamide N-methyltransferase (NNMT) regulates nicotinamide adenine dinucleotide (NAD+) and methionine metabolism by catalyzing methylation of nicotinamide using S-adenosylmethionine as the methyl donor. It was reported that NNMT is abundant in kidney and its association with the age-related disease including chronic kidney disease was reported [32, 33]. Matrix metalloproteinase 7 (MMP7) has been studied for its potential clinical utility as a diagnostic tissue and prognostic serum biomarker for renal fibrosis and declining kidney function, with its levels measured using the SomaScan platform from serum samples or RNA sequencing from kidney tissues [34]. In addition, overexpression of MMP7 was reported to be associated with collagen deposition and fibrosis in the kidney through measuring level of MMP7 from serum samples [35]. Notably, our data demonstrate that there is an increase of TIMP3, NNMT and MMP7 expression in the aging renal tubulointerstitium in humans through proteomics. Further, we observed altered expressions of understudied proteins with aging including PRG4, CHST14 and SAP. The major known function of proteoglycan-4 (PRG4) is lubrication of joints in the musculoskeletal system [36]: however, its function in the kidney has not been investigated. Similarly, although the deposition of serum amyloid P-component (SAP) has been reported to be associated with aging in the glomeruli [37], its specific role in the kidney remains unclear. Carbohydrate sulfotransferase 14 (CHST14), which is involved in the synthesis of dermatan sulfate, a type of glycosaminoglycan, has been detected in the kidney, but its association with aging remains unclear [38]. Further investigation is required to elucidate the role of these proteins in the aging kidney.

Some of proteins that were observed to be downregulated in the old kidneys also play important roles in renal function. For example, Phenazine biosynthesis-like domain-containing protein (PBLD) is known to be involved in the inhibition of NF-kB signaling. A decrease in its levels may contribute to dysregulated inflammation, which could accelerate stress and aging [39]. Similarly, solute carrier family 23 member 1 (SLC23A1) is involved in reabsorption and excretion of vitamin C [40]. The age-related decline in vitamin C levels has been attributed to a decrease in the expression of vitamin C transporters [41]. Glycerol kinase 3 (GK3P) and glycerol kinase 2 (GK2) were also observed to be decreased. Functional enrichment of differentially expressed proteins revealed glycerolipid metabolism as a dominant KEGG pathway for downregulated proteins in the old group (Supplementary Table 3). Although altered lipid metabolism was identified in study of aging kidneys of mice [42], the role of glycerol kinases has not been investigated, which requires further investigation. Overall, our study provides insight into protein changes associated with renal aging at a spatial resolution, which will benefit future studies in the field.

## Materials and methods

### Case identification

Patients who underwent a radical nephrectomy with surgery dates between 2010 and 2019 were identified for analysis as previously described [44]. Immediately after nephrectomy, the kidney is sectioned to identify the tumor for histological analysis and to isolate tumor-free tissue from regions distant from the tumor site. Only patients with < 5% interstitial fibrosis and tubular atrophy (IFTA) and < 5% globally sclerosed glomeruli (GSG) were included for selection. It is well known that both glomerulosclerosis and IFTA progressively increase with age in the kidney parenchyma [44]. These age-related changes are readily identifiable on standard kidney histology and the sclerotic/fibrotic lesions are certainly associated with specific proteins [6, 7]. For our study, we were interested in age-related changes in the proteome of the histologically normal appearing cortical tubulointerstitium rather than the proteome of the final sclerotic/fibrotic lesions. Thus, we limited our proteomic analysis to individuals with < 5% GSG and < 5% IFTA. We further used LCM to sample only non-IFTA regions of the tubules and interstitium, excluding glomeruli as they will have a different proteome. Review of the kidney histology revealed no neoplastic findings. Patients with specific renal diseases such as a glomerulonephritis that could be a paraneoplastic syndrome were not included. Patients were divided based on age, with patients under 40 years at the time of surgery included in the “young” cohort and patients aged 70 or older at the time of surgery were included in the “old” cohort.

### Laser capture microdissection and trypsin digestion of tubulointerstitial samples

Kidney wedge sections away from the tumor were made into FFPE tissue blocks that were serially sectioned at 10 μm onto polyethylene naphthalate membrane slides. Deparaffinization was performed using three consecutive 5 min immersions in xylene followed by two changes in 100% ethanol and one change in 95% ethanol. The tissue sections were baked after placed on slides to ensure adherence and examined at the end of deparaffinization to confirm that no tissue was lost. After deparaffinization and rehydration, the specified tissue areas were laser cut using a PALM Robo Microbeam scope (Carl Zeiss Microimaging GmbH, Jena, Germany). A total surface area of approximately 20 mm^2^ of tubulointerstitial tissue was laser pressure catapulted into the caps of 0.5 ml tubes in 35 µl of 100 mM Tris pH 8.2/0.005% Zwittergent 3–16. The tubes were centrifuged to bring down the captured tissue sections, and proteins were extracted by heating the tubes at 98 °C on a ThermoMixer C (Eppendorf) with a heat conductive top to limit evaporation. Disulfide bonds were reduced with 5mM dithiothreitol (DTT) heated at 60 °C for 30 min. After cooling to ambient temperature, proteins were alkylated with 15 mM iodoacetamide for 30 min in the dark. 5 mM DTT was added to scavenge excess IAA. Enzymatic digestion was performed with the addition of 1 µg of LysC/trypsin mix (Promega) and incubation/mixing at 37 °C at 900 rpm for 18 h. The digested peptide mixtures were acidified to 1% trifluoroacetic acid (TFA), transferred to a 96 well PCR plate and desalted on a AssayMap Bravo (Agilent Technologies) using the 5 µl C_18_ cartridges (5190–6532, Agilent). The peptide concentration for each sample was determined using 5% of the eluted volume with the fluorescent peptide assay kit (23290, Thermo Fisher Scientific).

### TMT labeling

Samples corresponding to 40 µg of peptide were transferred to a 0.5 ml tubes and lyophilized in a spinning vacuum concentrator. The dried peptides were solubilized in 40 µl of 100 mM triethylammonium bicarbonate, (pH 8.5), and each sample was mixed with 100 µg of a unique TMTpro reagent (A44520, Thermo Fisher Scientific) solubilized in 10 µl of acetonitrile. After incubation for 1 h at room temperature the reactions were quenched with 5 µl of 5% hydroxylamine and a 2 µl aliquot from each sample was pooled and analyzed by tandem mass spectrometry to ensure the labeling efficiency was greater than 98%. The samples were then pooled to match the reporter ion intensities from each channel and the mix diluted to 4 ml in 0.1% TFA/5% acetonitrile. Samples were desalted using solid phase extraction with a Sep Pak Plus C_18_ cartridge (Waters) and the eluted TMTpro labeled peptides were lyophilized.

### Basic pH RPLC fractionation

To reduce the sample complexity, the dried TMT labeled peptide mixture was solubilized in 500 µl 10 mM ammonium formate, pH 8.5 and separated into 96 fractions using a Dionex ultimate 3000 RS HPLC system with a XBridge BEH column (C_18_, 4.6 mm x 250 mm, Waters). The system was set up with 10 mM ammonium formate; pH 8.5 in water for the A solvent and 10mM ammonium formate; pH 8.5/90% acetonitrile for the B solvent. The separation gradient was 5% B to 60% B over 60 min followed by a 2-minute jump to 80% B while maintaining a constant flow rate of 0.5 ml/minute. The 96 fractions were concatenated to 24 fractions and lyophilized.

### Mass spectrometry data acquisition

The peptide fractions were analyzed by nanoLC-tandem mass spectrometry using an Exploris 480 Orbitrap mass spectrometer coupled to a Ultimate 3000 RSLCnano HPLC system (Thermo Scientific) with 0.1% formic acid in 98% water/2% acetonitrile for the A solvent and 0.1% formic acid in 80% acetonitrile/10% isopropanol/10% water for the B solvent. Each fraction was solubilized in 0.1% formic acid and pumped onto a Halo C_18_ 2.7 μm EXP stem trap (Optimize Technologies, Oregon City, OR) with 0.1% formic acid/0.05%TFA at a flow rate of 8 ml/minute. The trap was placed in line with a 35 cm x 100 μm PicoFrit (NewObjective) spray tip columns manually packed with Acclaim C_18_, 2.2 μm particle size stationary phase and the peptides separated with a gradient of 3–35% B over 120 min at a flow rate of 400 nl/minute. The mass spectrometer was set for data dependent acquisition with a 3 s cycle time. The MS1 survey scan range was from 340 to 1800 m/z at resolution 120,000 (at 200 m/z) and the AGC set for a maximum of 2 × 10^6^ ions and a 50 ms ion injection time. Ions in the scan range of 340–1400 m/z with positive charge states from 2 to 5 were sequentially selected for high energy collisional dissociation fragmentation MS/MS scans at resolution 45,000 with a NCE setting of 32 and the isolation width set to 0.7 m/z. Fragment ions were scanned from the starting mass of 110 m/z. The MS2 AGC setting was 200% (2 × 10^5^ ions) and the maximum ion injection time was set to auto. The dynamic exclusion feature was used to prevent ions selected for MS2 and any ions within an m/z of 7 ppm from being selected for fragmentation for 30 s. Two peptides (GLLFEVGDAVEGLELTEATPGYR and VTIQMLTQSLEEVVR) were synthesized by the Mayo Clinic Proteomics Core to validate their identification. Approximately 5 pmol of synthetic peptides were labeled with 250 µg of TMT reagent (135 N channel) and analyzed using Exploris 480 Orbitrap mass spectrometer in data dependent acquisition mode.

### Protein identification and quantitation

The mass spectrometry raw data files were searched with Andromeda against the SwissProt human database in MaxQuant (version 1.6.17), setup for MS2 reporter ion quantification with TMTpro 16-plex isobaric labels. Parameters were set for full trypsin specificity with oxidized Met and N-term protein acetylation allowed as variable modifications and carbamidomethyl cysteine as a fixed modification. Mass tolerances were set at 4.5 ppm for MS1 and 20 ppm for MS2 and protein identifications with single peptide minimum were filtered at 1% FDR at the peptide and protein level. Protein TMT quantitation was performed using an inhouse R-script to calculate fold-changes and p-values. Reporter ion channel correction factors were applied to PSMs which were then filtered to exclude peptides exceeding the threshold maximum of 50% isolation interference. Sample groups missing 50% of values were removed for comparisons with no imputation applied. Sample normalization was by median subtraction and t-test comparisons made with protein level log_2_ ratios.

### Statistical analysis

Numeric features were summarized with means and standard deviations; categorical features were summarized with frequency counts and percentages. Protein expression data were log2 transformed prior to analysis. All sixteen samples were processed in a single batch, removing the need to account for batch effects. Similarly, the distribution of protein expression levels did not differ between samples; thus, no normalization procedure was required. Multivariate analysis of differential expression between the old and young cohorts was performed using moderated regression adjusted for patient sex and hypertension status. The transformed expression data were compared between the old and young cohorts using moderated t-tests. These moderated t-tests combine the protein-specific variance with assay-wide variance under a Bayesian approach to provide more robust estimates of the standard errors under low sample sizes. Results from these tests were reported as log-fold changes for differential expression in the old cohort relative to the young cohort. P-values were adjusted for multiple comparisons using a tail area-based false discovery rate correction, and significant differences were declared at an adjusted p-value threshold of 0.05 (i.e., FDR < 0.05). Functional enrichment analysis for differentially expressed proteins was performed using DAVID [45].

## Conclusions

The ability to resolve morphologically distinct microstructures spatially at a high resolution have enabled substantial understanding into the tissue-specific phenotype. Our work demonstrates the suitability and the benefits of deploying a mass spectrometry-based multiplexed labeled proteomics approach to analyze microstructural morphology of laser microdissected FFPE tissue at spatial resolution. Importantly, proteomics of microdissected kidney samples has been mostly focused on glomeruli. To the best of our knowledge, this is the first study not only to identify proteins playing an important role in kidney function but also to quantify changes in protein expression in the aging tubulointerstitium at spatial resolution with in-depth proteome coverage. Further it also offers an insight for translation into globally applicable immunohistochemical tests and potential prognostic biomarkers useful for predicting renal disease outcomes, which were discovered using highly clinically relevant FFPE samples. One limitation of the study is the small sample size, which requires further studies on larger cohorts. A higher multiplexing methods using TMT 35-plex reagents [46] can be employed along with automated workflow for LCM and sample preparation to enhance throughput and data robustness [25, 43].

## Electronic supplementary material

Supplementary Material 1. A summary of clinical metadata of patients used in the study

Supplementary Material 2. A summary of identified proteins from young and old patients

Supplementary Material 3. A summary of Gene Ontology and pathway enrichment analyses for differentially expressed proteins

Supplementary Material 4. Distribution of iBAQ value with proteins highlighted mentioned in the study

## Data Availability

The mass spectrometry proteomics data have been deposited to the ProteomeXchange Consortium via the PRIDE partner repository with the dataset identifier PXD059176 [47].
