# Supplementary material for: Spatial proteomics to discover aging-associated alterations in the renal tubulointerstitium
Source: Clin Proteomics. 2025 Oct 8;22:37. doi: 10.1186/s12014-025-09550-8 (PMC12509392; doi:10.1186/s12014-025-09550-8)
Supplement: Supplementary file 4 — Supplementary Material 4. Distribution of iBAQ value with proteins highlighted mentioned in the study [file 12014_2025_9550_MOESM4_ESM.pdf]

Supplementary Information

Spatial proteomics to discover aging-associated alterations in the renal tubulointerstitium

Dong-Gi Mun[1], Ganesh P. Pujari[1], Gunveen Sachdeva[1,2], Benjamin J. Madden[3], M. Cristine Charlesworth[3], Kenneth Johnson[3], Luisa Ricaurte Archila[1], Mariam P. Alexander[1], Aleksandar Denic[4], Aidan F. Mullan[5], Vidit Sharma[6], Nicholas B. Larson[5], Anthony C. Luehrs[5], Andrew D. Rule[4,7*], Akhilesh Pandey[1,2,8*]

[1]Department of Laboratory Medicine and Pathology, Mayo Clinic, Rochester, MN 55905, USA
[2]Manipal Academy of Higher Education, Manipal, 576104, Karnataka, India
[3]Proteomics Core, Mayo Clinic, Rochester, MN 55905, USA
[4]Division of Nephrology and Hypertension, Mayo Clinic, Rochester, MN 55905, USA
[5]Division of Clinical Trials and Biostatistics, Mayo Clinic, Rochester, MN 55905, USA
[6]Department of Urology, Mayo Clinic, Rochester, MN 55905, USA
[7]Division of Epidemiology, Mayo Clinic, Rochester, MN 55905, USA
[8]Center for Individualized Medicine, Mayo Clinic, Rochester, MN 55905, USA

*__Corresponding authors__

Correspondence should be addressed to the following

Akhilesh Pandey, M.D., Ph.D.
Professor
Department of Laboratory Medicine and Pathology, Mayo Clinic
200 First Street SW, Rochester, MN 55905
Phone: 507-293-9564
Email: pandey.akhilesh@mayo.edu

Andrew Rule, M.D.
Professor
Division of Nephrology and Hypertension, Mayo Clinic
200 First Street SW, Rochester, MN 55905
Phone: 507-774-6182
Email: rule.andrew@mayo.edu

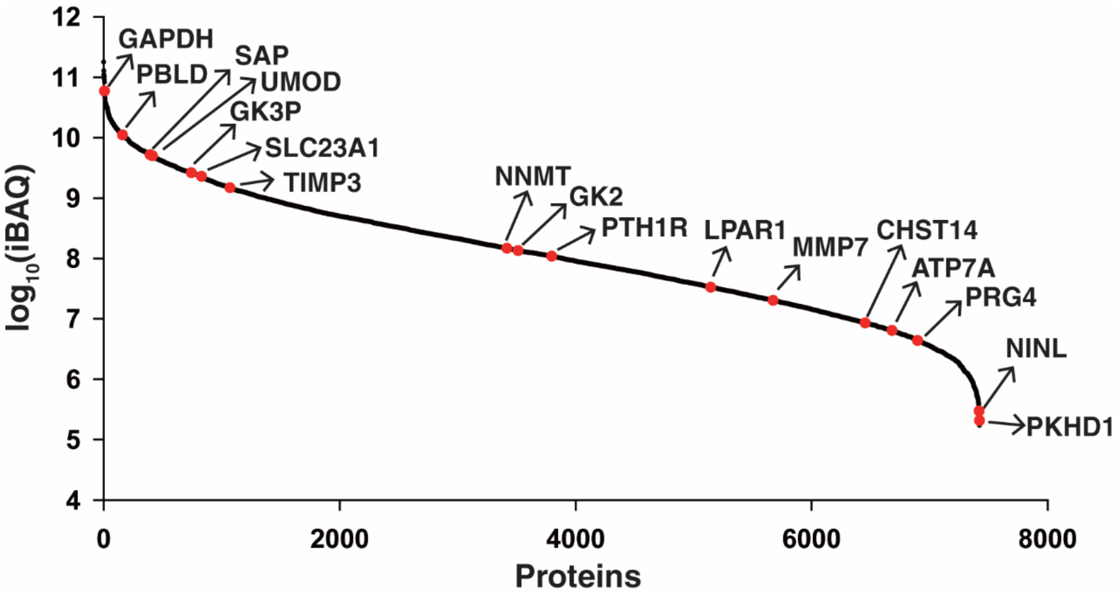

Supplementary Figure 1. Distribution of iBAQ value with proteins highlighted mentioned in the study
